# Barriers, Facilitating Factors, and Intersectoral Collaboration for Promoting Active Mobility for Healthy Aging—A Qualitative Study within Local Government in Germany

**DOI:** 10.3390/ijerph18073807

**Published:** 2021-04-06

**Authors:** Tanja Brüchert, Paula Quentin, Sabine Baumgart, Gabriele Bolte

**Affiliations:** 1 Institute of Public Health and Nursing Research, Department of Social Epidemiology, University of Bremen, Grazer Str. 4, 28359 Bremen, Germany; t.bruechert@uni-bremen.de (T.B.); sabine.baumgart@tu-dortmund.de (S.B.); 2 Health Sciences Bremen, University of Bremen, 28359 Bremen, Germany; 3 Faculty of Spatial Planning, Department of Urban and Regional Planning, TU Dortmund University, August-Schmidt-Straße 10, 44227 Dortmund, Germany; paula.quentin@tu-dortmund.de; 4 ARL—Academy for Territorial Development in the Leibniz Association, 30179 Hannover, Germany

**Keywords:** active mobility, age-friendly environment, healthy aging, intersectoral collaboration, local government

## Abstract

The promotion of walking and cycling to stay active and mobile offers great potential for healthy aging. Intersectoral collaboration for age-friendly urban planning is required in local government to realize this potential. Semi-structured interviews were conducted with the heads of planning and public health departments in city and district administrations of a Metropolitan Region in Germany to identify factors influencing action on the cross-cutting issue of active mobility for healthy aging. Although some administrations are working on the promotion of active mobility, they consider neither the needs of older people nor health effects. A lack of human resources and expertise, mainly due to the low priority placed on the issue, are described as the main barriers for further strategic collaboration. Furthermore, the public health sector often focuses on pathogens as the cause of morbidity and mortality, reducing their acceptance of responsibility for the topic. Facilitating factors include the establishment of new administrative structures, projects with rapid results that create awareness and credibility among citizens and politicians, additional staff with expertise in health promotion, and political commitment. In the future, new administrative structures for intersectoral collaboration are needed in order to consider various perspectives in complex developments, such as healthy aging, and to benefit from synergies.

## 1. Introduction

In the coming decades, the world will face the major demographic trend of aging. Germany is no exception, currently having one of the highest proportions of elderly people aged 65 years and older in the whole European Union [1]. Increasing life expectancies suggest that governments should consider successful and healthy aging as an important public health topic. For healthy aging, including the prevention of loneliness, it is important to stay active and mobile [2]. Active transport modes, such as walking and cycling, can support an active lifestyle. These activities substantially contribute to population health and life expectancy [3] and are crucial in the maintenance of autonomy and independence in later life [4,5]. The built environment can either promote or hinder active transport, and it can affect social participation and independence, e.g., by providing safe, aesthetically pleasing, and connected routes for walking and cycling that are separated from car traffic, as well as places and spaces to meet and rest [6,7,8]. Especially older adults with increasing mobility impairments face problems getting outdoors because of obstacles in their immediate neighborhood. However, the design of the built environment or decisions on traffic management are largely outside the sphere of responsibility of the public health sector. Moreover, the relevant actors are not always aware of health impacts of their decisions [9,10]. For example, measures to improve traffic flow, e.g., by widening road space, often unintentionally lead to more traffic or higher speeds because drivers feel safer. Consequently, such a measure may in fact hinder older pedestrians and cyclists from using road space and can further affect the health of vulnerable groups by increased noise and air pollution [11] and an increased risk of road accidents [12,13].

As the nexus of health, an active lifestyle and the built environment have been researched extensively in the last decades; translating these insights into municipal action is becoming a crucial next step. Governments and decision-makers should take action to connect the relevant policy fields through cross-cutting objectives such as the promotion of active mobility. Walking, cycling, and using public transport all have co-benefits in a range of policy fields, from air quality and economy, to health and equity [14]. In line with the Health in All Policies (HiAP) approach of the WHO [15], which calls for the consideration of health impacts at all levels of policy-making, the promotion of active mobility among the older population requires a vision and approach that crosses sectoral boundaries [14,16].

Leppo and Tangcharoensathien [17] and the WHO [18] emphasize the leading role of the public health sector in HiAP initiatives. Yet, the responsibilities, organizational structures, and power of public health institutions differ around the world. Public health institutions in Germany are established on the national, state, and municipal levels. The municipal level is of major interest in this study, as it is at this level that the implementation of (planning) measures takes place. The major tasks of municipal public health services are defined by federal state law. Although the concrete responsibilities vary according to the legislation of the individual states, there are specific focuses that are found in all legislation, albeit to differing degrees: (1) health protection (especially in terms of hazard prevention), (2) health promotion, (3) health care, and (4) health monitoring. The responsibility for health promotion has been defined by law only recently after the turn of the millennium (e.g., Lower Saxony was one of the last states in Germany to implement this legislation, doing so in 2007). The contribution of public health services to planning issues and the collaboration between planning and public health departments is only foreseen in some state laws, e.g., in North Rhine-Westphalia [19].

Intersectoral collaboration between planning and public health departments historically aims for better hygiene monitoring and the reduction of harmful exposure to environmental stressors such as air pollution and noise. Despite the broad consensus amongst different institutions and researchers worldwide for the need of intersectoral collaboration [20], little is known about whether and how intersectoral collaboration is taking place at the local level. This study explores the views of representatives of public health and planning departments in city and district administrations in a Metropolitan Region in Northwest Germany regarding their role in the cross-cutting issue of active mobility for healthy aging. The aim is to identify barriers and facilitating factors for action on the promotion of active mobility and healthy aging and for intersectoral collaboration. Understanding the institutional framework, resources, and operating principles of local public health and planning departments is essential for translating research findings of cross-cutting issues into administrative practice [21,22].

This research was conducted as part of the project AFOOT—Securing urban mobility of an aging population within the framework of the prevention research network AEQUIPA—Physical Activity and Health Equity: Primary Prevention for Healthy Aging [23]. The AFOOT project pursues an inter- and transdisciplinary approach to identify entry points for health and equity assessment in urban planning procedures, with a particular focus on small- and medium-sized towns in Germany [24]. This paper is based on a qualitative study design with problem-centered interviews of experts [25]. The heads of the public health and planning departments in a Metropolitan Region in Northwest Germany were interviewed to explore the views of the local public health and planning departments on the responsibilities for physical activity promotion, current activities, and collaborations between the two sectors as well as decision-making processes. After describing the applied methods, we present the results by levels of influence highlighting interrelationships of its components to address the cross-cutting issue of active mobility promotion among elderly in the public health and planning sectors. The paper concludes with a discussion on the implications for enabling and enhancing intersectoral collaboration on a cross-cutting issue such as active mobility for healthy aging.

## 2. Materials and Methods

### 2.1. Study Design

This research applies a qualitative study design based on problem-centered interviews of experts [25]. The heads of the public health and planning departments in a Metropolitan Region in Germany were interviewed as part of the project *AFOOT*—*Securing urban mobility of an aging population* [24]. These interviews had the primary aim of exploring the views of the local public health and planning departments on the responsibilities for physical activity promotion, current activities, and collaborations between the two sectors as well as decision-making processes.

### 2.2. Study Area and Sample

The study area is the Metropolitan Region of Bremen-Oldenburg in Northwest Germany. The AEQUIPA prevention research network [23] was established to strengthen regional networks and built capacities in this region. The AFOOT project is part of this prevention research network, and therefore, the expert interviews were conducted in this region. The Metropolitan Region encompasses five cities and eleven rural districts. Overall, 2.7 million people live in in this region of which 21.6% are 65 years and older. Walking and cycling for transport and for leisure is common in this area due to the mainly flat landscape. In the largest city studied (Bremen), the share of pedestrians and cyclists among all road users is 35% [26]. In the more rural areas of the study area, the dependence on cars is higher [6]. A recent survey of older adults in the small and medium-sized towns in the study area showed that 54% of the participants walk for transport and 41% cycle for transport at least 3 days a week [7,27].

To allow an overview over the entire Metropolitan Region, the sampling unit of this study included the heads of the planning and public health departments in the city and district administrations. Representatives of the approximately 120 local municipalities within the rural districts were not included in this stage of the AFOOT project. Firstly, the district representatives were supposed to know about current activities and developments also in the local municipalities and, secondly, have substantial influence on a more strategic and agenda-setting level. Nevertheless, it needs to be kept in mind that responsibilities in the study area are divided into district-level and municipal authorities. Public health services are located at the district level, while spatial planning competencies are divided into regional planning at the district level and land-use planning in the local municipalities. The city administrations, constituting districts in their own right, have both planning and public health responsibilities in their administration.

### 2.3. Data Collection and Analysis

From June to September 2015, the heads of the planning and public health departments from all 11 districts and all five city administrations in the Metropolitan Region were firstly contacted by email and then by telephone and asked for an expert interview. With the representatives who agreed to take part in the study, semi-structured expert interviews were conducted, which followed an interview guide (see Appendix A, Table A1). The interviews were structured into five sections: (1) Current situation of pedestrians and cyclists in the city/rural district, (2) Points of contact in one’s own work with the health and urban space, (3) Intersectoral collaboration, (4) Questions about whether there is a special project or concept in the city/rural district, and (5) Outlook. Each topic contained one or two main questions. Several control questions were used to specify the answers if necessary. The structure was supposed to allow for a natural conversation flow, while also ensuring that all relevant topics would be brought up [28]. The basic structure was designed for joint interviews with an expert of both public health and spatial planning, to give both actors the possibility to bring in their own perspective on what was said. In District 4, City 2, and City 3, the structure was slightly modified to meet a divergent constellation of experts or individual interviews.

The interviews lasted one hour on average. To represent the perspectives of public health and urban planning at each interview and to improve validity by Investigator-Triangulation, the interviews were led together by two scientists with different professions (T.B., P.Q.). The participants gave verbal consent. After starting the recording, the participants introduced themselves by their work history, their current position, and how long they had held this position. Audio from the interviews was recorded and transcribed verbatim with ©f4transkript software (Edu-Version v5.70.2, audiotranskription, Marburg, Germany).

Then, the transcribed material was analyzed by means of structuring content analysis [29] with deductive and inductive categories using the ©MAXQDA software (Version 12.3.6, VERBI Software GmbH, Berlin, Germany). The deductive categories were taken from the interview guide, while the inductive categories were derived by an open coding system on nearly half of the material by two scientists (T.B., P.Q.). Based on the method of consensual coding [30], the two scientists came together in the next step, checked matching passages, and discussed different codes until consensus was reached. Furthermore, categories were combined and systematized into a coding frame that made the category definitions more precise. The categories were defined and equipped with quotes, so that the remaining material could be coded [29]. Finally, similarities and differences in the experts’ statements on reasons for action or inaction on the cross-cutting issue of active mobility for healthy aging were identified and documented by typical statements. Comparability of the interviews is given by the interview guide and the institutional–organizational context that the interviewees have in common [31]. The reference to the statements in the results section names the department, the authority (numbered), and the paragraph in the transcript [32]. The interviews were conducted in German. The statements of the interviewees in this paper were translated by the authors.

## 3. Results

Seven out of eleven districts and three out of five city administrations agreed to take part in the interviews. There were 24 participants in eleven interviews, including three individual interviews and eight with two or more people (Table 1). If the heads of planning departments were unable to participate in the interview, some sent staff members from their department. If the heads of public health departments were indisposed, they did not send any representatives, although in some cases, they recommended other people or departments that they thought might have more expertise in the field. Altogether, non-participation was mainly justified with not being able to give substantial information on the topic, e.g., a person new to the position or not having the expertise, or a lack of time and personal capacities due to a high workload in other areas (especially accommodation and care for refugees in summer 2015). In one case, participation was not permitted by a superior.

**Figure 1:**
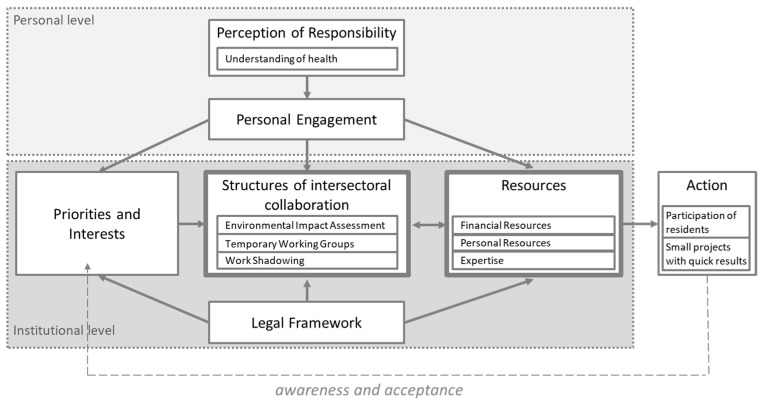
Levels of influence and interrelationships of its components on addressing the cross-cutting issue of active mobility promotion among elderly in the public health and planning sectors.

The reported views of the public health and planning officials on addressing the cross-cutting issue of active mobility promotion among elderly are here subsumed under two levels of influence: (1) the institutional level, which represents the direct work environment with given responsibilities by the legal framework, priorities, and interests of different stakeholder groups, administrative structures, and resources, and (2) the personal level, whereby the individual heads of departments and their staff are the focus, concentrating on their perceived responsibility and personal engagement. Each level of influence contains several components incorporating barriers and facilitating factors that are explained individually in the next paragraphs. Figure 1 comprises the findings to visualize the levels of influence and interrelationships of its components.

### 3.1. Institutional Level

#### 3.1.1. Legal Framework

The legal framework was an important reference point for the interviewees and often mentioned as it formally regulates tasks and responsibilities. The heads of the public health departments referred mainly to the state law of the public health service in Lower Saxony (NGöGD) from 2007. In terms of health promotion, §4 NGöGD states: *“The rural districts and cities support and coordinate preventive and health-promoting measures; they can also carry them out themselves.”* (translation by the authors). Among the interviewees, there were opposing opinions on the introduction of this section of the law: one public health official valued it positively, because health promotion got the legislative support to be a statutory task rather than a voluntary one (Public Health, District 6, 43), making it easier to call for action on health promotion topics. In contrast, another interviewee criticized this paragraph for being too vague, giving *“no room for maneuver”* because it is too unspecific (Public Health, District 7, 29); for this interviewee, it was impossible to specify a job description to ask for additional staff based on this section of the law. Furthermore, a head of the public health department in a city stated that the state laws of the public health services in general give *“little legislative back-up”* in decision-making processes compared to, e.g., regulations on noise exposure in the field of nature protection. Quote 1 in Table 2 illustrates how powerless some representatives of the health sector feel about implementing health promoting measures as they are not explicitly supported by law.

With regard to intersectoral collaboration, the legal framework of spatial and environmental planning was brought up by interviewees in terms of the opportunities it offers. In some formal planning procedures, such as environmental impact assessments, this legal framework foresees the involvement of public agencies—of which the public health department can be one. In these cases, the public health departments are asked to write statements on potential health effects. However, the statements submitted vary depending on the topic and scale. Statements were longer when health effects were obviously to be expected (e.g., hygienic compliance of a company), and shorter in overarching plans (e.g., land use planning). Others do not seem to use the opportunity to write a statement at all. Additionally, participation in these formal planning procedures was not seen as opportunity to draw attention to health-promoting aspects, such as the impact on older adults’ walking behavior. The statements of the public health services were mostly limited to harmful effects, which reflect their lack of perception of responsibility for health-promoting topics.

#### 3.1.2. Priorities and Interests

The heads of the public health and planning departments in the rural districts were aware of the growing number of elderly people and the resulting problems in mobility for older adults. Nevertheless, the topic was not regarded to be the most important one—in transport planning, the focus was still considered to be on individual automobility (Planning, District 6, 19), while in urban planning, the priorities were climate protection and adaptation (Planning, District 6, 52). In the public health department, the most important subjects for decades have been children and youth health (e.g., school entry health examination and vaccination). Older people only recently received more attention in terms of healthcare provision (Public Health, District 6, 52).

The recognition of the public health competence and of health in general was reported as a facilitating factor for priority-setting by some heads of public health departments. Some health representatives felt that their opinions are respected, but others noted that the public health issue has little weight in decisions (see Table 2, Quote 2). Therefore, networking both inside of the administration (e.g., with other departments) and outside of the administration (e.g., with politicians and citizens) was seen as important for strengthening the position of the department and to introduce new topics such as active mobility promotion (Public Health, District 2). In cases that lacked legal support, public health departments sought scientific evidence to support their position and to convince decision-makers by, for example, using the statements of the German Advisory Council on the Environment, which is an independent group of experts giving advice to the federal government (see Table 2, Quote 3). The reference to the statements conferred a certain importance and political support for their demands.

Challenges in priority-setting in the planning field mostly resulted from dealing with the interests of different stakeholder groups, i.e., other departments, politicians, and residents. Competing interests evolved e.g., in the case of transport planning, when car advocates compete with more “*innovative forces*” that want to foster public transport and cycling (Planning City 2, 40). Conflicts evolve also around the priority of inner urban development for short distances vs. the protection against noise. In economically prosperous parts of the study area, land allocation in general (e.g., for agriculture, industry, living, and recreation) is conflicted as the demand for land exceeds the available development area (Planning, District 5, 70). Thus, priority-setting is always a subject of different (sectoral) legislation or conceptual objectives: a decision in favor of one measure always means a decision against another. In addition, the implementation of political goals negotiated at federal level, such as the expansion of wind energy, can meet great resistance at the local level, e.g., from residents, which makes acceptance more difficult (see Table 2, Quote 4). This also has implications for political support. In the opinion of one head of a public health department, there were no priorities set on a long-term meaningful strategy in their rural district, because this would require courageous advocates who take innovative approaches, even when facing resistance. However, he admitted that such advocates are rare because of the risk of losing votes (see Table 2, Quote 5).

Nevertheless, priorities and decisions that are based on other priorities than health, such as economic considerations, could already have positive health impacts that were not yet seen. In several rural districts, the construction of new cycle paths resulted from tourism development (Planning, District 1, 19 and Planning District 5, 11). Such measures offer potential interfaces between public health and planning. However, in our study, there was no strategic cooperation on age-friendly infrastructures so far.

#### 3.1.3. Structures of Intersectoral Collaboration

Administrative structures specify the framework in which action is taken. Several heads of the public health departments complained about a lack of structure of intersectoral collaboration to foster new topics such as active mobility promotion. On one side, administrative structures are influenced by the given legal framework. One head of a public health department expressed his frustration at the missed legal opportunity on prevention and health promotion to establish new structures for public health services, meaning that new initiatives are hard to realize (see Table 2, Quote 6). On the other side, collaboration can take place at the working level. However, there were no established working groups or strategic collaboration structures on the topic of active mobility promotion by design in our study region. In some rural districts, the two sectors acted jointly in formal planning processes or on emerging topics, which attracted the attention of the population or the politicians, e.g., the establishment of medical service (Public Health, District 1, 74 and Public Health, City 1, 26).

The benefits of collaboration in terms of increasing awareness to hitherto unknown topics such as health could already be observed in larger projects, where working groups were installed temporarily, e.g., for the state funded project “Healthy Region” (District 6, 97), the statutory Regional Plan (District 1, 87–88), or a covenant on demography (District 4, 73–74). One planner described how he was *“sensitized”* and *“recognized the necessity”* of the topic of demography which lead to action (Planning, District 4, 74). The same planner suggested work shadowing during the educational phase in the administration in order to increase the mutual understanding of other areas such as public health (Planning, District 4, 57).

In addition to the need for structures of intersectoral collaboration in the district administration, the planners in District 1 and District 7 recommended giving advice and having periodic working groups with the local municipalities because the decision on construction projects is a local responsibility. Another planner suggested establishing a position in the rural administrative districts to advise local municipalities in terms of active mobility promoting environments for the elderly, or to develop a strategy, a concept, or guiding principles. However, this would require additional resources as the staff resources are scarce (District 3, 107).

In the end, several interviewees urged the need for new administrative structures so that important future challenges can be faced. However, they admitted that fundamental changes are rarely achieved overnight and would need long-term strategies that the decision-makers must be willing to approach (see Table 2, Quote 7). The planner in District 4 saw a facilitating factor to achieve structural change in growing *“social pressure and societal need“* that urges authorities to act and install supporting collaboration structures (Planning, District 4, 50). Therefore, setting priorities can directly influence structural changes.

#### 3.1.4. Resources

Action in terms of cross-cutting issues in our study region depended above all on existing resources. The lack of personnel resources and therefore a lack of time was repeatedly mentioned as a barrier to the public health departments in working on additional topics besides fulfilling their established statutory tasks, e.g., infectious disease prevention (Public Health, District 2, 56; Public Health, District 3, 57) (see Table 2, Quote 8).

The staffing for the topic of health promotion was poor in rural districts compared to city administrations. Moreover, in the case of prioritization in the public health department, savings were always first made in health promotion. This is due to the fact that a failure in this field would not result in any legal or even life-threatening consequences, when compared to e.g., hygiene monitoring (Public Health, City 1 and Public Health, District 3, 57). However, this attitude may result from the low priority of health promotion in general. Even in windows of opportunity to integrate a health perspective into other areas, such as statements in environmental impact assessments, the time for extensive new revisions and adaptations was lacking (Public Health, District 3, 61).

Financial resources for new projects played a less important role in the study region, as they can be compensated by project funds if necessary. Only in one case did the low financial status of the rural district hinder the application for funding due to the lack of the required co-funding. However, even in the city administrations, resources were mainly invested into established measures, such as self-help groups, because active mobility for healthy aging was seen as too specific a topic (Public Health, City 3, 51 and 72). This might be because of the general focus of physicians on the individual, rather than population-based solutions. In Germany, the heads of public health authorities are physicians with a special training in public health. Due to the legal framework, the main responsibilities of the public health service for decades have focused on hazard prevention, which is why there is a lack of expertise in the field of health promotion and overarching topics. Although the public health departments in the rural districts have had the possibility to engage in formal planning processes by writing statements, often the knowledge on cross-cutting issues was seen as not being so profound that someone could derive demands from it (Public Health, District 7, 35). One head of a public health department emphasized the dilemma in attempting to overcome this problem by joint discussions due to a lack of expertise on one hand and a lack of time on the other (see Table 2, Quote 9).

The same interviewee saw the problem in the training of the heads of public health departments, which focuses on administrative tasks. To overcome this lack of expertise in health promotion, one head of a public health department proposed to recruit graduates with a Bachelor or Master degree in public health because of their training in *“public relations, the community, the impacts on the whole of society”* (Public Health, District 3, 105). Another interviewee believed that these positions could also strengthen health promotion topics by networking and generating evidence (Public Health, City 1, 40).

### 3.2. Personal Level

#### 3.2.1. Perception of Responsibility

Beyond the responsibility given by the legal framework, the question about the perception of responsibility for a cross-sectoral topic such as active mobility promotion and the need to act arose from the interviews. The awareness of the health effects of active mobility modes was less apparent to heads of the public health departments than the planning departments. Most of the heads of the public health department saw no need for action on active mobility promotion from their own institution, as they saw the individual responsible for their own physical activity. These comments raise the question about the general understanding of health promotion by the public health services. Although health promotion is a statutory task similar to prevention, it was described as being *“still new ground”* and seen by a head of the public health department as more a societal and political topic in general rather than an issue of the public health services (Public Health, District 5, 52). In most administrations, health hazards had priority (Planning, District 4, 48; Public Health, District 5, 52) (see Table 2, Quote 10). In terms of cross-cutting issues, noise and air quality are the main environmental topics, followed by electromagnetic radiation as prevention of a hitherto unknown health hazards.

In almost all administrations, a more comprehensive understanding of the possibilities of health promotion were not often associated with the term “health”. Health was understood, in rather mono-dimensional terms, as the avoidance of illness. Therefore, projects based on “health promotion in settings” were already being implemented, whereas initiatives with a focus on “health-promoting settings” were still lacking. This can also be seen in the question of whether the public health service fostered the consideration of health and needs of vulnerable groups in planning processes. This was either allocated to the responsibility of the planning department (Public Health, City 3, 23, and 70) or was understood as an overarching goal whereby, for example, the good quality of new cycle paths serves everyone. Furthermore, older people were not seen as a *“specific target group”* to consider (Public Health, District 1, 29). Collectively, some heads of the public health and planning departments remarked that the topic of active mobility for healthy aging is quite complex and multifaceted and there is no *“panacea”* (Planning, District 6, 65) to tackle this topic with a specific intervention. One head of a public health department would not know where to begin (see Table 2, Quote 11).

#### 3.2.2. Personal Engagement

Apart from the statutory tasks, some heads of public health departments engaged in additional activities to bring in current or important topics. They saw themselves as service providers and preferred to work on issues that give *“pleasure”*—resulting in higher quality of work (Public Health, District 6, 45). One head of a health department was convinced that he must take the initiative and share his vision in order to gain the support of others (see Table 2, Quote 12). Additionally, if the head of the public health department in District 3 saw obvious health hazards, he took great care to write a comprehensive report, even if this was not required or time resources were scarce (see Table 2, Quote 13).

To put new approaches on the agenda, several heads of public health and planning departments were convinced that it is necessary to raise awareness at the outset for the vision and to *“make your voice heard”* (Planning District 1, 250). One head of a public health department tried to achieve support by being involved in larger projects to *“give the whole approach the appropriate value”* (Public Health, District 1, 135). Individual commitment seems to be a catalyst in agenda-setting processes and initiating activities.

### 3.3. Action

The issue of active mobility for healthy aging was not dealt with jointly by the authorities examined in our study region. However, participation of the population and small projects with quick results were mentioned as beneficial strategies to raise awareness and convince politicians to support new ideas and initiatives.

Several interviewees were convinced that courageous planning in the long-term is needed for innovations. It takes time to change the opinion “*in the minds of people*” (Planning, District 6, 21) on a special topic such as taking away space from cars to change it into space for active transport modes such as cycling. This was particularly poorly understood in more rural areas, where the lifestyle largely remains more car-oriented (Planning District 7, 134). Sustainable and meaningful planning does not only require time but also supporters and appropriate administrative structures (Public Health, District 1, 261). As it is more difficult to persuade private actors to invest and remain involved in long-term projects (Planning, District 6, 53 and 65), some heads of public health and planning departments were convinced that simple approaches with quick results have the greatest effect and are best accepted by the general population (Planning District 6, 46; Public Health, City 1, 50) (see Table 2, Quote 14).

Furthermore, it was seen as important to involve local stakeholders and residents by asking about their needs because standardized strategies and interventions often do not fit (Planning, District 6, 65). One planner explained that it can also help to inspire and convince people about the benefits of a certain topic or idea so that they become active themselves or support a special idea, e.g., by future workshops (see Table 2, Quote 15).

## 4. Discussion

To ensure population health in the 21st century, the Health in All Policies (HiAP) approach of the WHO calls for the consideration of health impacts at all levels of policy-making [18]. This requires a vision and approach that crosses sectoral boundaries. In our study, the issue of promoting active mobility for healthy aging through urban development is as an example of a cross-cutting topic that is not addressed jointly by the public health and planning departments. Although some authorities are working on the promotion of active forms of mobility, they consider neither the needs of older people nor health effects. While the focus of this study was not on evaluating a specific policy implementation, the results regarding barriers and facilitating factors for action and operation of intersectoral collaboration to foster active mobility for healthy aging presented here are mainly in line with other results of public policy research in the context of health promotion. The policy process is described as a complex system in which the relationships between the components are not always linear [33,34,35]. This qualitative study, which covers only two sectors in an entire administrative system, extends the picture by providing an initial approach to the complexity of addressing the cross-sectoral issue on a local level in Germany. The influencing factors for action and its relationships are summarized in Figure 1. Overall, the lack of structures for intersectoral collaboration, personnel resources, and expertise are cited as the main barriers for not addressing active mobility for healthy aging. However, these factors seem to be the result of the low priority of the topic in general. This is due to two main factors: legal requirements, whose implementation has been institutionalized over the years, and the responsibilities interpreted and perceived by the heads of health and planning departments.

Most public health professionals regarded the legal framework as not being supportive for the uptake of health promotion measures in its present form. Other states in Germany, e.g., in North Rhine-Westphalia [19], have already implemented collaboration between the departments of planning and public health in their state laws to foster action on the social determinants of health. Still, although the legal framework may be supportive, it is only one factor in the entire policy-making process. Despite the poor legal framework in our study region, one interviewee tried to substantiate his demands by using supportive scientific evidence. The literature emphasizes the importance of evidence for intersectoral collaboration, too [36]. On the other hand, Parkhurst [34] cautions to think carefully about how to implement research into policy and to be critical about the consequences, as one should not be under the misapprehension that the research results will be used in their own interest or used at all. Research has to be at least relevant and readable [22]. However, as was also brought up by a head of a public health department in our study, there is no reason to assume that one politician or policy actor would give up their position for a *“meaningful strategy”* or scientific evidence [34]. Riley and Nazelle [37] argue in a similar direction that the role of competing interests of different stakeholders can have a greater influence on the uptake of new topics than hard evidence.

The role of conflicts of interests and priorities was also discussed by our interviewees. Fostering physical activity is only one issue among many challenges that cities and governments are facing worldwide. Raising awareness in the population, under politicians and inside the municipality, was often mentioned as a core strategy in our study for gaining the support of new approaches. The participation of the population for increasing public acceptability and to share one’s own vision are also reported as a promising approach in the review by Nieuwenhuijsen et al. [38]. In comparison, Molnar et al. [39] argue that raising awareness alone does not lead to a buy-in for integrating health into non-health sectors in the long-term. “Win–win” strategies, on the other hand, facilitate implementation by highlighting benefits and thus increasing acceptance in all sectors.

However, no uptake of such new strategies will happen if the public health sector does not feel responsible for health promotion topics [36]. The results on the perception of responsibility and its relationship with priority-setting in this study is consistent with findings from South Australia about adopting an equity perspective by an HiAP approach. In their study, van Eyk et al. [40] identified the following barriers: (1) dominance of economic goals is prioritized over social policy ideas, (2) equity is not seen as core business, (3) there is a focus on lifestyle/individual behavior in strategies, and (4) there is a lack of shared understanding of equity. In our study, the understanding of health was rather reduced to disease prevention instead of a more comprehensive understanding that would include well-being and the importance of social interaction. This has also been observed in other countries [36,41,42]. Although a broad body of literature exists about the relationships between (active) mobility and health [14,18,43], our interviewees mostly did not make the link to the concept of the social determinants of health. An explanation could be that the profession of physicians working in the public health service mainly have training in disease prevention and individual health behavior, rather than health promotion meaning that expertise is lacking. Furthermore, the responsibility of the public health sector for health promotion has only been defined relatively recently in Germany; structures and routines are geared to familiar tasks because little has been done to address this new function, e.g., by hiring additional staff.

In addition, the responsibility for the topic of active mobility for healthy aging was rejected because of its complexity—the interviewees did not know where to start. As Exworthy [35] states, the complexity of social determinants of health makes them hard to act on in conventional policy-making environments. Jansson et al. [42] explain rejections of responsibility with the lack of clarity regarding the responsibility for health promotion by other sectors. Although it is widely argued that collaboration structures bundle resources in the long-term [18], the co-benefits seem to have not been realized in the studied departments. All interviewees consistently stressed the extent to which they are already fully occupied with established duties, confirming other research which points to the fear of additional work by HiAP approaches [36]. However, the interviewees were able to give insight into the ways in which one can adapt one’s practice to act within the legal framework and also advance issues. An engaged and passionate staff would make a profound difference between a measure being successful or not.

Finally, the importance of short-term projects was linked to setting priorities in the administration. Rudolph et al. [44] call such activities “low-hanging fruits” as early wins encourage future investments of time or other resources. Short-term projects were seen as a possibility to raise awareness and gain support [39], although participants were very aware of the need for long-term strategies to meet future challenges. However, one should be aware that individual behavior-oriented activities conducted at local levels are less effective in promoting population health [45,46].

### Strengths and Limitations

As a result of the voluntary nature of participation in the interviews, there might be a selection bias to the extent that participants were either more interested in the topic or had a greater knowledge on the issue from the beginning. Therefore, the interviewees might be more committed by nature. A complete survey of all administrations was intended, but only ten out of sixteen administrations participated. Non-participation was mainly justified by staff shortage and perceived lack of expertise in the field, which is consistent with the actual findings. Therefore, it can be assumed that statements from participants and non-participants would not differ to a great extent. Similar justification for non-participation was reported in a study from the UK with planners and public health practitioners on addressing obesity [41].

When interpreting the results, it should also be considered that the situation of paired interviews may have influenced the statements of the interviewees, which may have resulted in information being withheld in order not to displease the other person. The relationship between the interviewees should be taken into account in future research. Another limitation is that only districts in Lower Saxony and a selection of cities were included in the study. These districts and cities are bound to state laws, which differ from other states in Germany. Therefore, comparability with other federal states in Germany, in the case of the influence of the state law, is limited. Nevertheless, this study is one of the few that focuses on the lowest level of administration where both sectors (urban planning and public health) can be found. Studies often focus on higher levels and policy-making rather than the practical implementation of regulations. However, the routines and practices of planning decisions at the municipal level in rural areas were not explored in this study. To research implementation processes in the future, the perspective of the municipal level should be considered when implementing an HiAP strategy.

## 5. Implications for Research and Practice

Based on our findings, we recommend defining leadership roles for health and intersectoral collaboration beyond health care and disease prevention. Responsibilities have to be clarified [44]. The WHO [18] attributes a key role to the public health service in HiAP. However, this in turn requires a high level of personnel resources and knowledge on the social determinants of health, which is particularly difficult to implement in smaller administrations. In our study area, there was little or no room to reallocate personnel resources. While structural change may be important (though difficult to achieve), communicating the win–win of adopting a “health lens” [47] in non-health sectors and to work within given structures, e.g., the framework of the formal planning procedures, may be a first starting point [48,49]. Therefore, we recommend expanding the expertise of the public health service staff by providing training on the social determinants of health, interdisciplinarity, communication, negotiation, and integrative thinking. Short handouts, guidelines, or policy briefs about research results that are clearly formulated and give advice could furthermore improve the transfer of evidence into practice [50,51]. However, it should be evaluated whether such efforts reach practice and lead to desired action, as the use of evidence in policy-making still depends strongly on (political) interests [34].

The potential role of the public health service in HiAP is currently a matter of debate in Germany [52,53]. Future research should evaluate such professions and roles in administrative routines together with researching policy-making processes in smaller administrations, e.g., by transition experiments [54]. Transition experiments aim at transformational change and innovation by collaboration between actors from academia and society (government, industry, and citizens).

In terms of agenda-setting, community participation appears to be important. Participatory engagement of communities is seen as an integral part of appropriate accountability and decision-making for complex health problems [38,55]. However, attention must be paid to the understanding of the term “health” in this area as well, and how it is questioned, so that the determinants of health in a broad sense can be identified [55].

Nevertheless, the basic approach, and probably the most crucial point for a sustainable consideration of health in planning processes, will be the commitment to health and well-being throughout the government [56]. Pinto et al. [57] found that a high-level commitment to intersectoral collaboration is even more important than funding for HiAP initiatives. The call for long-term changes in structures by our interviewees was seen to require courageous decisions and directions of the decision-makers. The longstanding low priority of health and resulting lack of structures in many countries were exposed by the COVID-19 pandemic in 2020/21. We will see how this pandemic affects the priority of public health in the long term.

## 6. Conclusions

In the future, it will become increasingly important to consider complex developments such as population aging and (healthy) living environments and the resultant tasks in the planning process at national, state, municipal, and community level. Researchers have to understand the operating principles, procedures, and structures in administrations if they want to initiate an HiAP strategy at a specific level. Active mobility as an example of an overarching topic has not yet been addressed with the aim of promoting health in the public health service at the municipal level of our study region. It has been shown that smaller administrations in particular lack the resources (especially personnel and expertise in health promotion) to address specific and cross-sectoral issues such as the promotion of active mobility in old age. Structures for intersectoral collaboration could bundle resources and thus contribute to the goal that all departments achieve their target requirements. However, such administrative structures essentially depend on the priority of the topic and the definition of leadership roles. Here, the state and federal governments are also in charge of providing legislation that foresees approaches that overcome sectoral boundaries to improve public health. Especially in smaller administrations, such approaches require additional staff with knowledge on the social determinants of health and HiAP. As long as the health and well-being of the population is not on the agenda, it will be difficult to progress complex and cross-sectoral issues. However, implementing this vision is only possible if decision-makers are not reluctant of new ways of thinking, making fundamental changes, and supporting innovative and forward-looking solutions that need time to adapt to.

## Figures and Tables

**Table 1:** Characteristics of interviewees.

	N	Sex/Gender	
		Female	Male
No. of interviewees *	24	4	20
Single Interviews	3	0	3 **
Double Interviews	5	0	5 **
Group Interviews (≥3 Interviewees)	3	3 **	3 **
Institutional age ***			
median (min,max)	8 (1–34)	8 (1–21)	12 (1–34)
**Position**			
Head of Public Health Department	8	0	8
Head of Planning/Transport Department	8	1	7
Administrative staff—Planning/Transport	4	1	3
Administrative staff—Health	0	0	0
Administrative staff—Seniors/Demography	3	2	1
Head of regional development department	1	0	1
**Administrative Level**			
No. of interviews in city administrations	4	0	4 **
No. of interviews in district administrations	7	3 **	7 **

* Total number of interviewees distributed over three individual interviews, five double interviews and three group interviews, ** number of interviews with participation of the respective gender, *** number of years working in this position.

**Table 2:** Illustrating quotations of interviewees by components.

Legal framework	
Quote 1	*“It is easier to protect an owl couple from [traffic noise] than an older couple living there.”* (Public Health, City 3, 74)
**Priorities and interests**	
Quote 2	*“So, we always try to underpin and strengthen our positions, but with health, we have a relatively poor position and often lose out when it comes to conflicting goals.”* (Public Health, City 3, 74)
Quote 3	*“It’s not law, but it exerts pressure if there are demands. What the Advisory Council says, that also has a political character.”* (Public Health, City 3, 74).
Quote 4	*“So, there are EXTREME resistances [of the population] against it and that also leads to the fact that the local authorities are sometimes torn between two positions. On the one hand, they have to keep an eye on the interests of their residents; on the other hand, they know very well that they also need to give space to these alternative energy sources.” (Planning, District 1, 10)*
Quote 5	*“Such politicians, who are willing to give up their careers for a long-term meaningful strategy—there are very few.” (Public Health, District 3, 138)*
**Structures of intersectoral collaboration**	
Quote 6	*“I believe that we also have to change structurally […] on these issues. That’s one of those great things I expected in the law of public health services, that they think about STRUCTURES. BUT, I actually realized they’re going on like they used to. And that is wrong. We can have so many great ideas, but we will fail at the smallest trivialities.”* (Public Health, District 1, 261)
Quote 7	*“[…] that is our biggest common problem, to get things into the heads in a forward-looking way, that we are ready to accept changes to which we haven’t been ready for years.”* (Planning, District 1, 267)
**Resources**	
Quote 8	*“In the end, it’s personnel resources ... I do not have to ask for [project] money, but I have to have the opportunity to take care of those [additional topics].”* (Public Health, City 1, 111)
Quote 9	*“We have neither the expertise nor the time, we have to read, we have to deal with it, we have to talk more often about such issues. That’s just not possible, that’s not possible.”* (Public Health, District 3, 61)
**Perception of responsibility**	
Quote 10	*“Here in the department, we work risk-oriented. We look at the risks that affect people in the city, and we are always concerned with minimizing those risks ... so when I talk about noise action planning, I’m talking about noise. I’m also talking about quiet zones, but I do not have the focus on physical activity in quiet areas or whatever else I can do because it’s all about minimizing that stressor noise, that’s our point of view, in that direction we argue.”* (Public Health, City 3, 17, and 21)
Quote 11	*“The more I think about the topic, promoting physical activity in old age is a quite complex topic which has many facets. Spontaneously I wouldn’t know where to start.”* (Public Health, District 6, 55)
**Personal Engagement**	
Quote 12	*“Work is not done by itself and sometimes, if the mountain will not come to Muhammad, choose the opposite way and just go to the people and say: ‘I have this vision, let’s get together and discuss how we can achieve that.’”* (Public Health, District 5, 110)
Quote 13	*“[...] it has to be a severe issue, that I’ll rack my brain over things that are not at all part of my routine.”* (Public Health, District 3, 83)
**Action**	
Quote 14	*“[...] that is the plea for very simple solutions that may not be the pure science and may not be particularly rewarded in planners circles especially [...], but in that case, it is a clarity that goes along with the fact that [...] the people […] just do it that way.”* (Planning, City 2, 57)
Quote 15	*“[...] So, if you want to bring such a topic into implementation, then you have to make it a topic again [...] so invite people and inspire them, ‘That’s a topic!’. And when they jump on it and are enthusiastic about the matter, then you can let go, then they march off. But that is difficult enough.”* (Planning, District 6, 58–59)

## Data Availability

The data presented in this study is not publicly available due to privacy issues.

## Appendix A

**Table A1:** Interview guide.

**Current situation**
How would you describe the situation for cyclists and pedestrians in your city/rural district?
● *Do you take into account the needs of special groups, e.g., older people?*
● *Are there any spatial differences?*
● *What kind of required action do you see from your own position?*
● *In which context do your work on this topic? Does physical activity matter?*
**Points of contact in one’s own work**
Are there any points of contact between health and spatial planning? Alternatively, what could you imagine?
● *Do you deal with prevention by environmental change?*
● *Are there any interventions on active mobility promotion?*
● *What about addressing health issues in the planning department?*
**Intersectoral collaboration**
How and when do the health department and the planning department work together? What experiences do you have?
● *If none, why?*
● *What experiences do you have with health impact assessment and environmental impact assessment?*
● *Are there any committees in which you work together? What is your experience? What goals did you achieve together?*
**Question about a special project or concept in the city/rural district**
Please tell us about this project/concept. How did it start and who is involved?
● *Who determined the content alignment? Who is the leader of the project/concept?*
● *How are the needs of older people addressed in the project/concept?*
● *How is the promotion of physical activity addressed in the project/concept?*
● *In what way did the health department participate in the development of the project?*
● *Are there any other projects on physical activity promotion where the health department is involved?*
**Outlook**
The topic of health promotion is now supported by the new law of prevention, also in the community setting. What chances do you see to better integrate the topic into your daily routine by this law? What conditions need to be complied with?
● *What role could the health and planning departments take up, respectively, in projects of healthy city development?*
● *Which other actors would be relevant?*
